# *Auxin Response Factor2* (*ARF2*) and Its Regulated Homeodomain Gene *HB33* Mediate Abscisic Acid Response in *Arabidopsis*

**DOI:** 10.1371/journal.pgen.1002172

**Published:** 2011-07-14

**Authors:** Li Wang, Deping Hua, Junna He, Ying Duan, Zhizhong Chen, Xuhui Hong, Zhizhong Gong

**Affiliations:** 1 State Key Laboratory of Plant Physiology and Biochemistry, College of Biological Sciences, China Agricultural University, Beijing, China; 2 China Agricultural University–Purdue University Joint Research Center, Beijing, China; 3 National Center for Plant Gene Research, Beijing, China; Peking University, China

## Abstract

The phytohormone abscisic acid (ABA) is an important regulator of plant development and response to environmental stresses. In this study, we identified two ABA overly sensitive mutant alleles in a gene encoding Auxin Response Factor2 (ARF2). The expression of *ARF2* was induced by ABA treatment. The *arf2* mutants showed enhanced ABA sensitivity in seed germination and primary root growth. In contrast, the primary root growth and seed germination of transgenic plants over-expressing ARF2 are less inhibited by ABA than that of the wild type. ARF2 negatively regulates the expression of a homeodomain gene *HB33*, the expression of which is reduced by ABA. Transgenic plants over-expressing HB33 are more sensitive, while transgenic plants reducing HB33 by RNAi are more resistant to ABA in the seed germination and primary root growth than the wild type. ABA treatment altered auxin distribution in the primary root tips and made the relative, but not absolute, auxin accumulation or auxin signal around quiescent centre cells and their surrounding columella stem cells to other cells stronger in *arf2-101* than in the wild type. These results indicate that ARF2 and HB33 are novel regulators in the ABA signal pathway, which has crosstalk with auxin signal pathway in regulating plant growth.

## Author Summary

Abscisic acid is a phytohormone that regulates many aspects in plant growth and development and response to different biotic and abiotic stresses. Research on ABA inhibiting seed germination, controlling stomatal movement, and regulating gene expression has been widely performed. However, the molecular mechanism for ABA regulating root growth is not well known. We have set up a genetic screen by using ABA inhibiting root growth to identify ABA related mutants and to dissect the molecular mechanism of ABA regulating root growth. In this study, we identified two new mutant alleles that are defective in *ARF2* gene. ARF2 is a transcriptional suppressor that has been found to be involved in ethylene, auxin, and brassinosteroid pathway to control plant growth and development. Our study indicates that ARF2 is an ABA responsive regulator that functions in both seed germination and primary root growth. ARF2 directly regulates the expression of a homeodomain gene *HB33*. We demonstrate that ABA treatment reduces the cell division and alters auxin distribution more in *arf2* mutant than in the wild type, suggesting an important mechanism in ABA inhibiting the primary root growth through mediating cell division in root tips.

## Introduction

Abscisic acid regulates many important aspects including seed development, dormancy, germination, vegetative growth, and plant responses to environmental stresses [1]. ABA is required for normal plant growth as ABA-deficient mutants reduce cell vigor and are usually smaller [2]. Different developmental stages of *Arabidopsis* seedlings exhibit different response to ABA. In the early germination stage for establishing embryonic axis, the seed germination and post-germination growth are more sensitive to ABA (during 48 hr after seed imbibition) than other stages and more than 3 µM ABA will block the germination and post-germination growth [3], [4]. Genetic screening during this stage has been performed and identified some specific ABA responsive factors such as ABA INSENSITIVE3 (ABI3) and ABI5, which play critical roles in regulating seedling growth mainly during seed germination and post-germination growth period [5], [6]. However, after more than 48 hr of seed imbibition, higher concentrations of ABA are needed to inhibit seedling growth [1]. Recent studies have identified four core components in the ABA signaling pathway, which include soluble PYR1/PYL/RCAR ABA receptors, PP2C phosphatases, SnRK2 kinases and ABA-responsive transcriptional factors for gene regulation or SLAC1 and other channels for regulating guard cell movement, indicating a relative simple and short regulating pathway [7]–[12]. The SnRK2 (sucrose non-fermenting 1-related protein kinase) triple mutants and the dominant *abi1-1* and *abi2-1* mutants show insensitive to ABA in all ABA responses including seed germination, seedling growth and guard cell movement [4], [13]–[15]. Low concentrations of ABA promote root growth through the promotion of the quiescent centre quiescence and the suppression of stem cell differentiation [16]. However, high concentrations of ABA can inhibit root growth through inhibiting cell division [17], [18]. Some DNA replication related mutants are hypersensitive to ABA in seed germination and seedling growth, suggesting that ABA signal might inhibit cell division through regulating the DNA replication related proteins [18].

In order to find the new genes in ABA response, we performed a genetic screen by using ABA inhibiting root growth phenotype [18]–[20]. Here we identified two *ARF2* mutant alleles that were hypersensitive to ABA in both seed germination and primary root growth. ARF2 directly regulates the expression of a homeodomain gene *HB33*. Our data indicate that ARF2 is a negative, and HB33 is a positive regulator in ABA mediating seed germination and primary root growth.

## Results

### *arf2* mutants are more sensitive to ABA than the wild type in both seed germination and root growth

The sensitivity of seed germination on ABA has been used to identify some classic ABA sensitive and ABA insensitive mutants [4]. In order to find more new ABA responsive mutants, we take advantage of root growth sensitivity to ABA as a selection standard. 5-day seedlings grown on MS were transferred to MS medium supplemented with 30 µM ABA, and the mutants whose root growth is slower than the wild type were selected after growing for 7 days. From an ethyl methyl sulfonate-mutagenized *Arabidopsis Columbia* M2 population [18], [20], we identified two mutants whose primary root growth is hypersensitive to ABA. Genetic analysis indicates that the two mutants were caused by different recessive mutations in the same gene. Because the two mutants showed similar ABA sensitivity and growth phenotypes, we selected one mutant, named *arf2-101*, for further analysis. This mutant was backcrossed four times with the wild type to remove other possible mutations. For map-based cloning of the *ARF2* gene, we used an F2 population obtained from a cross of *arf2-101* with the *Arabidopsis Landsberg* accession. In *ARF2/AT5G62000*, a G-to-A transition in *arf2-101* was identified at the splice junction of the 8th exon and 7th intron, and this transition would produce a premature protein. In another mutant, *arf2-102*, a TGG-to-TGA (G1125→A1125) transversion of *ARF2* was identified in codon 375, and this transversion would cause premature translation termination. The phenotypic characteristics of *arf2-101*, which included dark green leaves, late flowering, abnormal flower morphology, partial sterility in early formed flowers, and delayed senescence, were similar to those described previously for other *arf2* mutant alleles such as *arf2-7* (a T-DNA insertion mutant from the *Arabidopsis* stock center) [21]–[23].

**Figure 1:**
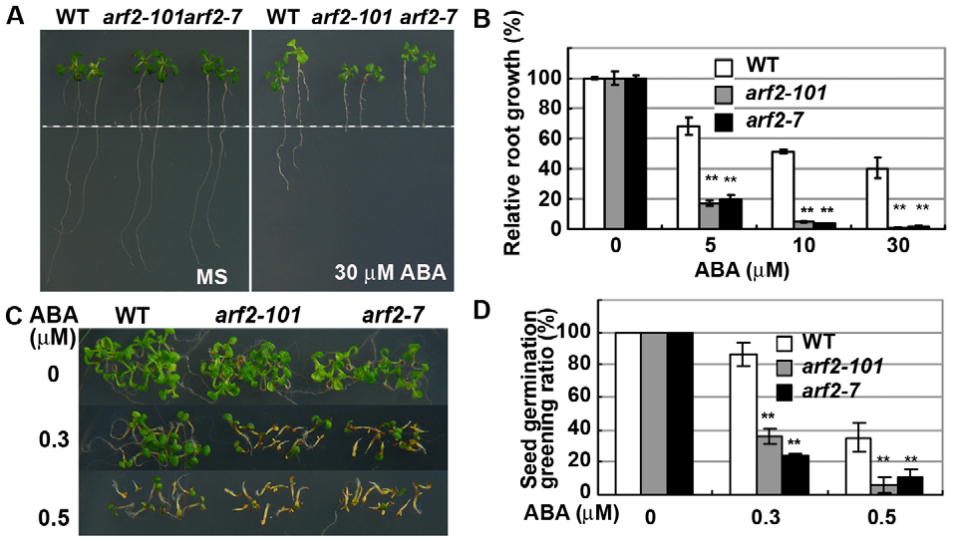
*arf2* mutants are hypersensitive to ABA. (A,B) Root growth of the wild type (WT), *arf2-101*, and *arf2-7* on MS medium containing different concentrations of ABA. Five-day-old seedlings grown on MS medium were transferred to MS medium containing 0 to 30 µM ABA and were grown for 7 days before being photographed and measured. The dot line indicates the places where the root tips were after just transferring. 30 seedlings were measured in each experiment. Relative root growth represents the root growth of seedlings after treatment with ABA comparing with that without ABA treatment. Three independent experiments were done. Values are means ±SD, **p<0.01. (C,D) Comparison of seed germination greening among the wild type, *arf2-101*, and *arf2-7* on MS medium supplemented with 0, 0.3 and 0.5 µM ABA. Three independent experiments were done. At least 30 seeds were accounted in each of three different plates for each time. Values are means ±SD, **p<0.01.

As primary root growth is much easier to compare than shoot growth, here we mainly focus on the primary root growth inhibition by ABA. We compared the primary root growth of *arf2-101* and the wild type with and without ABA treatment; *arf2-7* was included for comparison. Seedlings grown for 5 days on MS medium without ABA were transferred to MS medium supplemented with different concentrations of ABA. On MS medium without ABA, primary root growth was similar for *arf2-101*, *arf2-7*, and the wild type (Figure 1A). Inhibition of primary root growth by ABA, however, was greater for *arf2-101* and *arf2-7* than for the wild type. At 5 µM ABA, the relative primary root growth was about 20% in *arf2-101* and *arf2-7*, but more than 65% in the wild type (Figure 1A, 1B). ABA at ≥30 µM almost completely arrested primary root growth of *arf2-101* and *arf2-7* but only inhibited 60% the wild-type (Figure 1A, 1B). In contrast, primary root growth did not differ between *arf2-101* and the wild type when the MS medium contained NaCl (from 50 mM to 150 mM, for salt stress), mannitol (200 to 350 mM, for osmotic stress), LiCl (15 to 30 mM, for ionic toxic stress), or the plant hormone jasmonate (JA), ACC (a precursor for ethylene), brassinosteroid (BR), or gibberellin (GA3) (Figure S1). We also compared the sensitivity of *arf2-101* mutant to ABA with the wild type during seed germination and post-germination stage (Figure 1C, 1D). At 0.3 µM ABA, about 40% of *arf2-101* and 22% of *arf2-7* showed seed germination greening (green cotyledon), while about 85% of wild type showed seed germination greening, indicating that *arf2* mutants are more sensitive to ABA than the wild type in the seed germination and post-germination.

Combined together, the results indicate that ARF2 is involved in two different ABA responsive stages, i.e. both the earlier stage during seed germination and later developmental stage of root growth.

### Expression of *ARF2* is induced by ABA, and transgenic plants over-expressing *ARF2* are more resistant than the wild type to ABA

**Figure 2:**
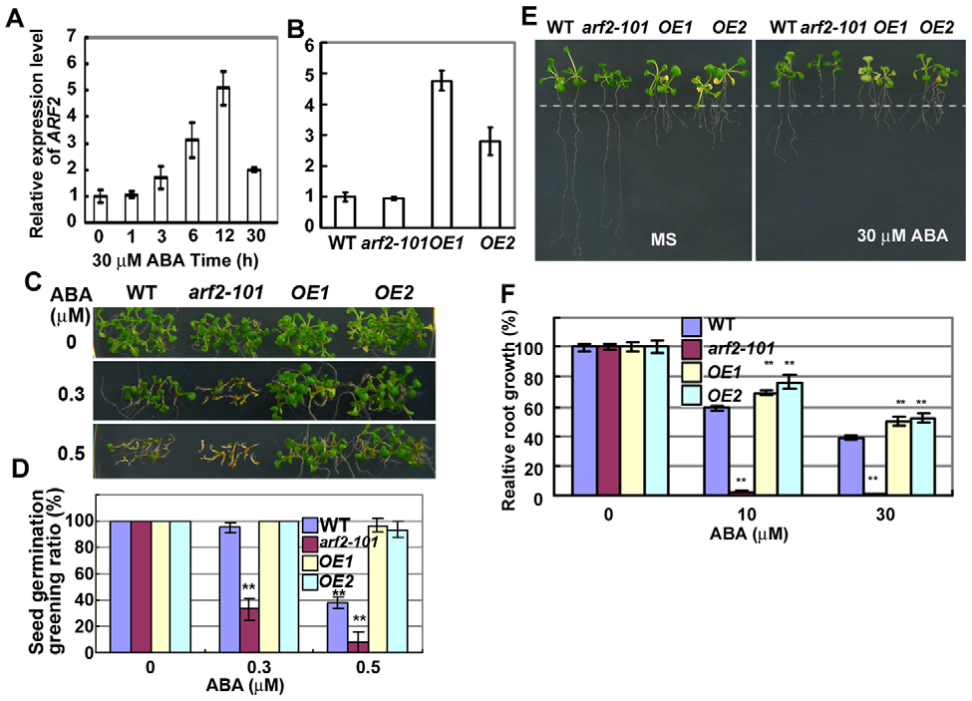
Overexpression of *ARF2* increases resistant to ABA. (A) Expression of *ARF2* was induced by ABA treatment. Total RNAs extracted from 14-day-old seedlings treated with 30 µM ABA for different times were reversely transcripted and used for qRT-PCR. *ACTIN* was used as a control. Three biologically independent experiments were done with similar results. The results shown were from one experiment with triple technical repeats. Values are means ±SD. (B) The relative expression of *ARF2* in transgenic plants over-expressing *ARF2*. The relative *ARF2* levels in two over-expressing lines, *ARF2-Flag OE1* (*OE1*) and *ARF2-Flag OE2* (*OE2*) with earlier flowering phenotype, were measured by qRT-PCR. *ARF2* from the wild type (WT) or *arf2-101* were used as controls. The *arf2-101* mutation did not affect itself expression. (C,D) Seed germination greening of WT, *arf2-101*, *OE1*, and *OE2* on MS medium containing different concentrations of ABA. Seeds of the different accessions were imbibed on MS medium plates with or without ABA at 4°C for 2 days before the plates were transferred to 20°C. Three independent experiments were done. At least 30 seeds were accounted in each of three different plates for each time. Values are means ±SD, **p<0.01. (E,F) Relative root growth of WT, *arf2-101*, and *arf2-7* on MS medium containing different concentrations of ABA. Five-day-old seedlings grown on MS medium were transferred to MS medium containing 0, 10 and 30 µM ABA and were grown for 7 days before being photographed and measured. The dot line indicates the places where the root tips were after just transferring. 30 seedlings were measured in each experiment. Relative root growth represents the root growth of seedlings after treatment with ABA comparing with that without ABA treatment. Three independent experiments were done. Values are means ±SD, **p<0.01.

To investigate the role of ARF2 in ABA responses, we first measured the effect of ABA on expression of *ARF2*. In the *ARF2* promoter region from −750 to −744, there is a reverse ABF/ABRE binding *cis*-element ( GCCACGT ) [24], suggesting that *ARF2* expression might be regulated by ABA response factor(s). Two-week-old seedlings were treated with 30 µM ABA for 0–30 h, and total RNAs were extracted and used for qRT-PCR. As shown in Figure 2A, ABA treatment increased the expression of *ARF2* (relative to the control without ABA, from about 5 folds at 12 hr to 2 folds at 30 hr). The ABA inducible expression further supports that ARF2 is involved in ABA response.

We next determined whether increasing the transcripts of *ARF2* influences the ABA sensitivity of the wild type plants. We constructed a super promoter-driven *ARF2* that is fused with a flag tag and transferred it to wild-type plants by *Agrobacterium*-mediated flower dip transformation. Although most transgenic plants overexpressing *ARF2-flag* showed the *arf2* mutant phenotype because of co-suppression as reported previously [23], several independent transgenic lines with high expression of *ARF2-flag* were obtained. We selected two of these independent high expression transgenic lines for further study. qRT-PCR analysis indicated that *ARF2* transcripts were more abundant in the two over-expressing lines than in the wild type or *arf2-101* (Figure 2B). As reported previously, two *ARF2-flag* over-expressing lines flowered earlier and showed leaf senescence earlier than the wild type [23], suggesting that the flag tag did not affect ARF2 function. We analyzed seed germination greening of the *ARF2-flag* over-expressing lines in response to ABA. Without addition of ABA (Figure 2C and 2D), seed germination greening ratio was similar for the wild type, *arf2-101*, and the two *ARF2* over-expressing lines. With increasing ABA concentration in the medium, however, seed germination greening ratio was much greater in the two *ARF2* over-expressing lines than in the wild type or *arf2-101*; *arf2-101* was the most sensitive to ABA. At 0.5 µM ABA, seed germination greening ratio is about 40% in the wild type, about 95% in two overexpressing lines and 10% in *arf2-101* (Figure 2D). *ARF2-flag* over-expressing plants had shorter primary roots, and more lateral roots than the wild type. However, the primary root growth of *ARF2* over-expressing lines was more resistant to ABA than the wild type (Figure 2E, 2F). These results indicate that ARF2 negatively regulates ABA inhibition of seed germination and primary root growth.

### ARF2 binds to the *HB33* promoter and regulates *HB33* expression

**Figure 3:**
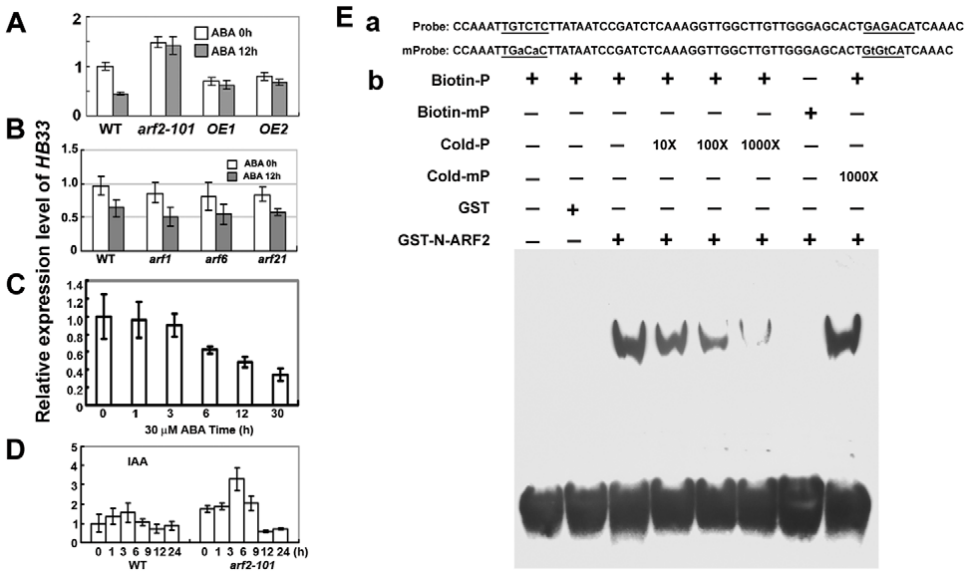
ARF2 binds to the *HB33* promoter and regulates *HB33* expression. (A) Expression of *HB33* was negatively regulated by ABA and by ARF2. Two-week- old seedlings of the wild type (WT), *arf2-101, ARF2-OE1*, and *ARF2-OE2* were treated with 0 or 30 µM ABA for 12 h. Total RNAs were extracted, reversely transcripted, and used for qRT-PCR. *HB33* expression in WT without ABA treatment was used as a standard for normalizing the relative expression level. Three biologically independent experiments were done with the similar results. The shown results are from one experiment with three technical replicates. Values are means ± SD, n = 3. (B) *HB33* expression was not affected by ARF1, ARF6 and ARF21. The similar experiments were done as in (A) by using *arf1*, *arf6* and *arf21* mutants. Three biologically independent experiments were done with the similar results. The shown results are from one experiment with three technical replicates. Values are means ± SD, n = 3. (C) Time course of *HB33* expression in WT. Two-week-old seedlings were treated with 30 µM ABA for different times. The relative expression of *HB33* was determined by qRT-PCR. Three biologically independent experiments were done with similar results. The results are from one experiment with three technical replicates. Values are means ± SD, n = 3. (D) The expression of *HB33* under IAA treatment. Two-week-old seedlings were treated with 5 µM IAA for different times. The relative expression of *HB33* was determined by qRT-PCR. Three biologically independent experiments were done with similar results. The results are from one experiment with three technical replicates. Values are means ± SD, n = 3. (E) Gel-shift analysis of ARF2 N-terminus binding to *cis*-elements in the promoter of *HB33*. a, The oligonucleotide sequences of probe and mutated form probe (mProbe) within the *HB33* promoter used in the EMSA. Underlined letters indicate the sequences of ARF2-recognition motifs (TGTCTC). mProbe: ARF2-recognition motifs in probe were mutated as indicated by small letters. b, Interaction between GST-N-ARF2 protein and biotin-labeled Probe and mProbe by SDS-PAGE analysis of purified GST-N-terminus ARF2 fusion protein. Purified protein (6 µg) was incubated with 25 fM biotin-labeled probe or mProbe (mP). For the competition test, non-labeled probe with different concentrations (from 10 to 1000 times) or labeled mP (1000 times) was added in the above experiment.

**Figure 4:**
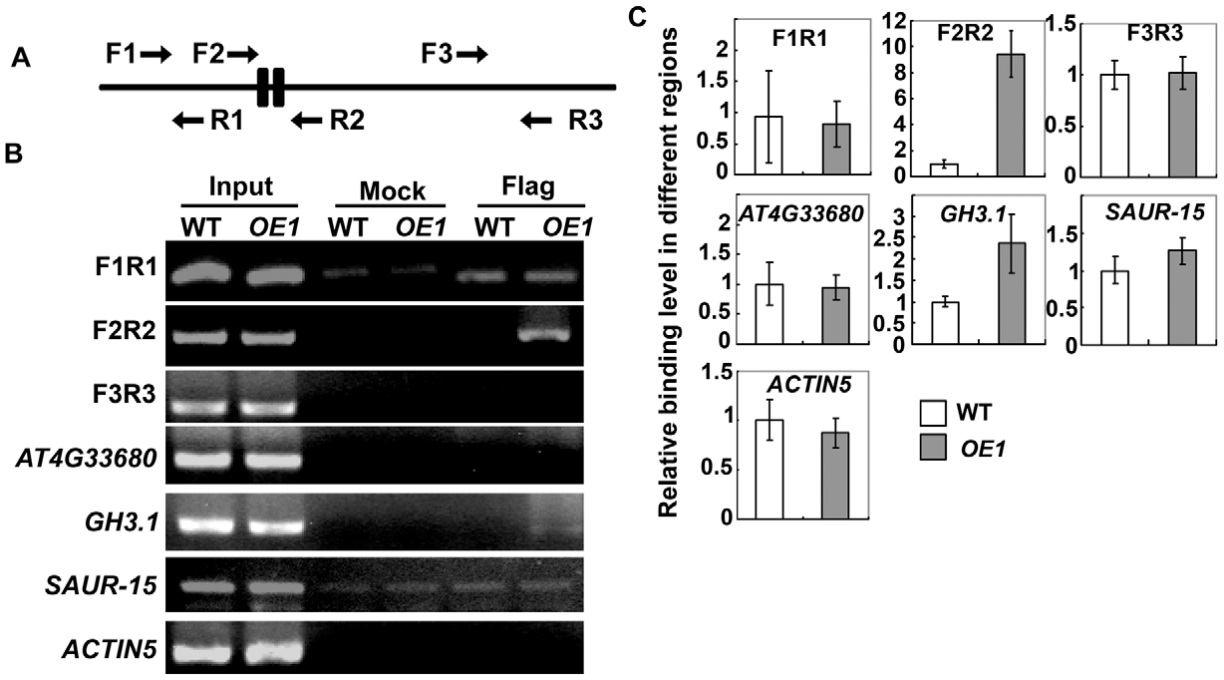
Chromatin immunoprecipitation (ChIP) assay on the promoter of *HB33*. (A) Three pairs of primers were used. Primer pair F2 and R2 covered the promoter region containing AuxRE *cis*-element. F1 and R1 pair of primers are upstream of AuxRE *cis*-element, and F3/R3 pair are in the coding region of *HB33*. (B) Chromatin immunoprecipitation (ChIP) assay on the promoter of *HB33*. One transgenic line (*ARF2-OE1*) overexpressing ARF2-Flag and flag antibody were used for the ChIP assay. The wild-type seedlings were used as the negative control. *ACTIN*, *AT4G33680*, *SAUR-15* and *GH3.1* were included in this experiment as controls. Three independent experiments were done with the similar results. Results from one experiment are shown. (C) qRT-PCR of ChIP assay in (B). Three independent experiments were done with similar results, each with triple biological repeats. Data were from one experiment with three technical replicates. Values are means ± SD, n = 3.

*arf2* mutants decrease transcriptional levels of three ethylene biosynthesis genes (*ACS* ) in flowers [21] and a senescence-related gene *SAG12* (*SENESCENCE ASSOCIATED GENE 12*) [22]. In both *in vitro* and *in vivo* assays, ARF2 negatively regulates the reporter genes under the control of a synthetic promoter with AuxREs (auxin-responsive elements) [23], [25]. In searching for genes regulated by ARF2 from microarray data [21], we found that *AT1G75240* encoding HOMEOBOX PROTEIN 33 (HB33) was up-regulated in the *arf2* mutant (the average signal was 153 in the wild type and 276 in *arf2-6* from three independent repeats) [21]. We confirmed the microarray results by qRT-PCR, which showed that the expression of *HB33* was about 1.5 times greater in *arf2-101* than in the wild type (Figure 3A). In order to know whether *HB33* is also regulated by other ARFs, we compared the *HB33* expression of the wild type with *arf1*, *arf6* and *arf21* mutant, and did not find the expression difference of *HB33* between these mutants and the wild type (Figure 4B), suggesting that *HB33* is regulated specially by ARF2. In contrast to the expression of *ARF2*, the expression of *HB33* was inhibited by ABA treatment in the wild type but not in the *arf2-101* mutant (Figure 3A). The reduction of *HB33* expression by ABA was further confirmed by a time-course experiment (Figure 3C). We also determined that the expression of *HB33* was lower in two *ARF2-flag* overexpressing lines than in the wild type and was not further reduced by ABA treatment (Figure 3A), suggesting that ARF2 represses *HB33* expression. It appears that auxin treatment weakly induced the expression of *HB33* in the early times in both the wild type and *arf2* mutant (Figure 3D). It is likely that ARF2 does not regulate *HB33* expression by auxin.

AUXIN RESPONSE FACTORs (ARFs) are transcription factors with a conserved N-terminal DNA-binding domain that binds to TGTCTC *cis*-elements in promoters of auxin-responsive genes [25]–[27]. In the *HB33* promoter region, we found two AuxREs (TGTCTC), one in the position −202∼−197 and the other in the reverse direction (GAGACA) in the position −157∼−152. ARF2 contains the N-terminal DNA-binding domain that targets the AuxREs without the help of a middle or C-terminal part (the middle region for transcriptional activation or repression, and the C-terminal dimerization domain) [25]. We expressed the GST fused with the DNA-binding domain in N-terminus (ARF2-N1-470) in *Escherichia coli* and purified the fused protein with the help of the GST tag. Gel-shifting was performed to test whether the recombinant protein could bind to the AuxREs. As shown in Figure 3E, a shifted DNA-binding band was detected with addition of GST-ARF2N1-470 and labeled DNA probes, but no band was detected in the GST control. When unlabeled DNA probe was increased gradually in the reaction mixture, the DNA-binding band was abolished. The GST-ARF2 protein, however, did not bind the mutated DNA probes (mP), and the mP did not compete with labeled DNA probes. These results suggest that the ARF2 N-terminal DNA binding domain binds to AuxREs in the promoter of *HB33*.

We then used the chromatin immunoprecipitation (ChIP) assay to test whether ARF2 could bind to the *HB33* promoter *in vivo*. In this experiment, we used one transgenic line over-expressing ARF2-Flag (OE1) and the wild type as a negative control. Flag antibody was used for ChIP analysis. As shown in Figure 4B, ARF2-Flag bound to the *HB33* promoter region, which contains two *cis*-elements as used in the Gel-shift assay (F2/R2), but could not bind to the gene encoding region (primer pair F3/R3) or to the promoter region that does not contain the AuxRE *cis*-element (primer pair F1/R1). qRT-PCR results were shown in Figure 4C for each pair of primers (F1R1, F2R2 and F3R3). In theory, all ARF proteins should have ability to bind the AuxREs. However, *in vivo*, each of ARFs only binds to specific AuxRE in the promoter regions of limited genes. In order to exclude no specific binding of ARF2 to the promoter region, we also included two genes, *SAUR-15* and *AT4G33680*, both of which contain the AuxRE *cis*-element in their promoter regions, but their expressions are not regulated by ARF2, and the gene *GH3.1* which contains the AuxRE *cis*-element in their promoter region and is regulated by ARF2 [28], as controls. ChIP assay indicated that ARF2 did not bind to the promoter regions of *SAUR-15* and *AT4G33680*, but bound to the promoter region of *GH3.1* (Figure 4B, 4C). The results indicate that ARF2 does not bind to all of the AuxRE *cis*-element regions. ARF2 binding to the promoter of *HB33* and *GH3.1 in vivo* might need the help of other components for its binding specificity [29].

### Transgenic plants overexpressing *HB33* have an increased sensitivity to ABA in primary root growth inhibition

**Figure 5:**
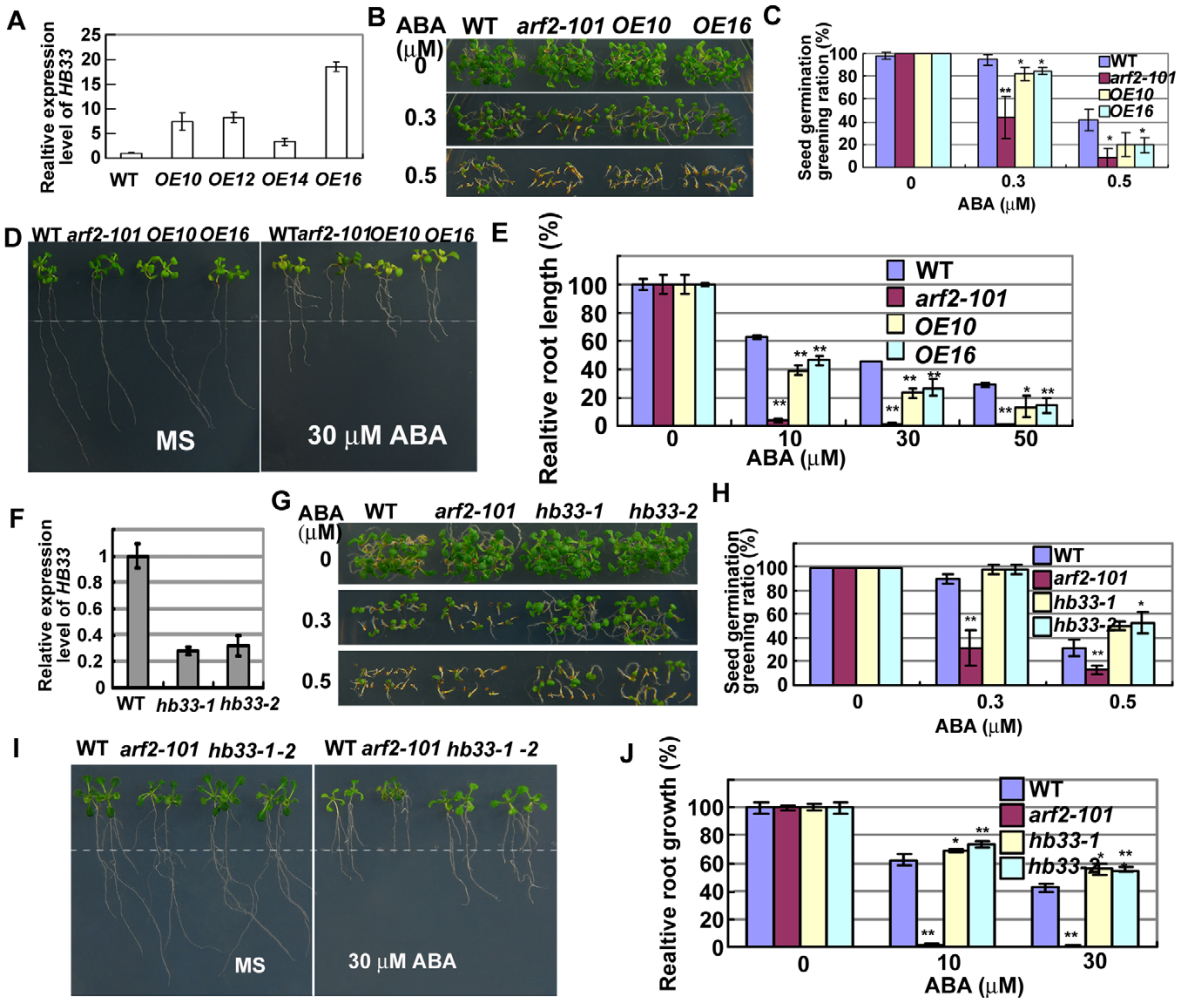
Adverse ABA phenotypes of transgenic plants overexpressing *HB33* and reducing *HB33* by RNAi. (A) The relative expression of *HB33* in different transgenic lines. The wild type (WT) was used as a standard control. *OE10* and *OE16* were selected for further study. (B,C) Comparison of seed germination greening among WT, *arf2-101*, HB33 overexpression line *OE10* and *OE16* on MS medium supplemented with 0, 0.3 and 0.5 µM ABA. Three independent experiments were done. Values are means ±SD, **p<0.01. (D,E) Comparison of relative root growth among WT, *arf2-101*, *OE10* and *OE16*. Five-day-old seedlings grown on MS medium were transferred to MS medium containing 0, 10, 30 and 50 µM ABA and were grown for 7 days before being photographed and statistics analysis. The dot line in (D) indicates the places where the root tips were after just transferring. 30 seedlings were measured in each experiment. Relative root growth represents the root growth of seedlings after treatment with ABA comparing with that without ABA treatment. Three independent experiments were done. Values are means ±SD, **p<0.01. (F) The relative expression of *HB33* in two RNAi transgenic lines (*hb33-1* and *hb33-2*). WT was used as a standard control. (G,H) Comparison of seed germination greening among WT, *arf2-101*, *hb33-1* and *hb33-2* on MS medium supplemented with 0, 0.3 and 0.5 µM ABA. Three independent experiments were done. Values are means ±SD, **p<0.01. (I,J) Comparison of relative root growth among WT, *arf2-101*, *hb33-1* and *hb33-2*. Five-day-old seedlings grown on MS medium were transferred to MS medium containing 0, 10, and 30 µM ABA and were grown for 7 days before being photographed and statistics analysis. The dot line indicates the places where the root tips were after just transferring. Relative root growth represents the root growth of seedlings after treatment with ABA comparing with that without ABA treatment. 30 seedlings were measured in each experiment. Three independent experiments were done. Values are means ±SD, **p<0.01.

HB33 in *Arabidopsis* belongs to a zinc finger-homeodomain (ZF-HD) subfamily containing 14 members that can dimerize with each other in a yeast two-hybrid assay [30]. Most proteins in this family do not have an intrinsic activation domain and might need to interact with other factors for transcriptional activation [30]. We made transgenic plants that over-expressed *HB33* under control of a super promoter [31], and qRT-PCR analysis confirmed the higher expression of *HB33* in these independent transgenic lines (Figure 5A). We selected two transgenic lines, *HB33-OE10* and *HB33-OE16*, for further study. We tested the seed germination sensitivity of two transgenic lines on MS medium without or with 0.3 or 0.5 µM ABA. As shown in Figure 5B and 5C, no difference in seed germination greening was found among wild type, *arf2-101* and two overexpression lines on MS medium without ABA. However, two overexpression lines were more sensitive to ABA than wild type, but less sensitive to ABA than *arf2-101* in seed germination. We further compared the effect of ABA on primary root growth of *HB33-OE10* and *HB33-OE16* lines. Five-day-old seedlings grown on MS medium were transferred to MS medium supplemented with different concentrations of ABA. With all of the tested ABA concentrations (10–50 µM), primary root growth of *HB33-OE10* and *HB33-OE16* was more sensitive than that of the wild type to ABA, although the sensitivity was still less than that of *arf2-101* (Figure 5D, 5E). These results indicate that HB33 overexpression exhibits similar ABA sensitive phenotypes as *arf2-101* in seed germination and primary root growth.

### *HB33* RNAi transgenic plants are more resistant to ABA than the wild type

It appears that ARF2 is a negative regulator, while HB33 is a positive regulator which is controlled by ARF2 in ABA signal pathway. We hypothesize that reducing HB33 would result in ABA resistance. We used a fragment of *HB33* which has low homologous sequence with other *HB* genes and made *HB33* RNAi transgenic plants. We took two (*hb33-1*, *hb33-2*) from several independent transgenic lines and checked *HB33* expression by qRT-PCR. Two RNAi lines had only about 25% *HB33* expression of the wild type (Figure 5F). We then compared the ABA sensitivity of *hb33-1* and *-2* in seed germination and primary root growth with that of the wild type and *arf2-101*. *hb33-1* and *hb33-2* were more resistant to ABA than the wild type or *arf2-101* in both seed germination and primary root growth (Figure 5G–5J). The results indicate that plants with reducing HB33 show similar ABA phenotypes as plants over-expressing ARF2. However, *arf2-101 hb33-1* or *arf2-101 hb33-2* double mutants showed ABA sensitive phenotype as *arf2-101* mutant (data not shown), indicating that reduced HB33 is required, but not sufficient for suppressing the *arf2* mutant phenotype in ABA signaling.

### ABA treatment reduces the expression of *CYCB1;1* promoter*-GUS* more in the *arf2-101* mutant than in the wild type

**Figure 6:**
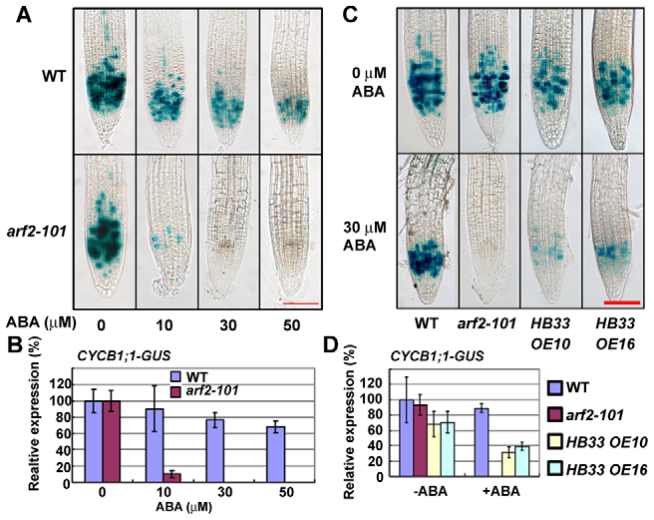
ABA treatment significantly reduces the expression of *CYCB1;1* in *arf2-101* and *HB33* overexpressing plants. (A) Promoter *CYCB1;1::GUS* expression in the wild type (WT) and *arf2-101* with 0, 10, 30 and 50 µM ABA for 36 h. (B) The intensity of GUS coloration was quantified by using Adobe Photoshop CS (Adobe Systems Inc.; San Jose, CA, USA) software. About 10 root tips were measured. The relative intensity without ABA treatment in WT or *arf2-101* is considered as 100%. (C) Promoter *CYCB1;1::GUS* expression in the wild type, *arf2-101* and HB33 overexpressing line 10 and 16 (*OE10* and *OE16*) with 0, or 30 µM ABA for 36 h. Red bar = 100 µm. (D) The intensity of GUS coloration was done as in (B). The relative intensity without ABA treatment in WT is considered as 100%.

Previous studies indicate that ethylene inhibits root growth by retarding cell elongation but not by affecting cell division [32], while ABA inhibits root growth by inhibiting cell division [17], [18]. CYCB1;1 is a G2/M marker protein that might be regulated by PLETHORA2 that is essential for root quiescent center (QC) establishment and stem cell maintenance [33], [34]. We compared *CYCB1;1* promoter-*GUS* expression between the wild type and *arf2-101*. Under normal growth conditions (i.e., when ABA was not added), *GUS* expression in the root tips did not clearly differ (Figure 6A, wild type; Figure 6B, *arf2-101*). Addition of different concentrations of ABA decreased GUS expression in root tips of both the wild type and *arf2-101*. However, the GUS expression level was decreased more in *arf2-101* than in the wild type. At 10 µM ABA, more GUS staining spots were observed in the wild type than in the *arf2-101*. ABA at ≥30 µM completely inhibited the GUS expression in the *arf2-101*, but not in the wild type (Figure 6A and 6B). The results indicate that *arf2-101* mutants are more sensitive to ABA inhibition of cell division than the wild type.

As plants over-expressing *HB33* exhibit a similar ABA sensitive phenotype as *arf2* mutant in root growth, we further checked the *CYCB1;1::GUS* expression pattern in the *HB33* over-expressing lines. As shown in Figure 6C and 6D, without ABA treatment, GUS expression was a little less in *HB33 OE10* and *OE16* than in the wild type or *arf2-101*. 30 µM ABA treatment for 36 h significantly reduced the expression of *CYCB1;1::GUS* in two *HB33* over-expressing lines (*OE10* and *OE16*). The results suggest that similar to *arf2* mutants, HB33 overexpressors are more sensitive to ABA inhibition of cell division than the wild type.

### Genetic interaction between *arf2* and the classic *abi* mutations

**Figure 7:**
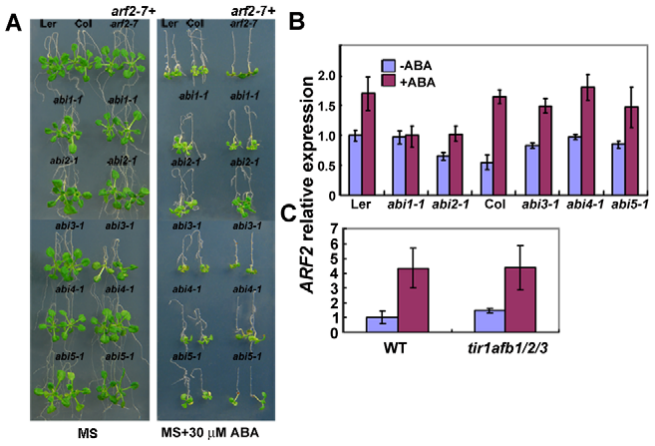
Genetic analysis of *arf2-7* with *abi* mutants. (A) Root growth of *arf2-7*, *abi1-1*, *abi2-1*, *abi3-1*, *abi4-1*, *abi5-1*, *arf2-7 abi1-1*, *arf2-7 abi2-1*, *arf2-7 abi3-1*, *arf2-7 abi4-1 and arf2-7 abi5-1* on MS medium or MS medium containing 30 µM ABA. Five-day-old seedlings grown on MS medium were transferred to MS medium or MS medium containing 30 µM ABA and grown for 7 days before being photographed. (B) The expression of *ARF2* in different *abi* mutants treated with or without 30 µM ABA for 12 h. Three biologically independent experiments were done with the similar results. The shown results are from one experiment with three technical replicates. Values are means ± SD, n = 3. (C) The expression of *ARF2* in the wild type (WT) and *tir1 afb1 afb2 abf3* mutant treated with or without 30 µM ABA for 12 h. Three biologically independent experiments were done with the similar results. The shown results are from one experiment with three technical replicates. Values are means ± SD, n = 3.

Many ABA-insensitive mutants have been identified by different screens in *Arabidopsis*. We crossed *arf2-7* (carrying a T-DNA insertion that can be easily used to identify a double mutant) with *abi1-1* and *abi2-1*, two dominant negative mutants, [4], [13], and *abi3-1*, *abi4-1* and *abi5-1* mutants [5], [6], [35], and obtained *arf2-7 abi1-1*, *arf2-7 abi2-1*, *arf2-7 abi3-1*, *arf2-7 abi4-1* and *arf2-7 abi5-1* double mutants. The homozygous double mutants were confirmed by both sequencing of the mutation sites and genetic analysis of F3 and F4 seeds for no segregation. With respect to ABA inhibition of root growth, *arf2-7 abi1-1* and *arf2-7 abi2-1* had an ABA-insensitive phenotype similar to that of the *abi1-1* and *abi2-1* single mutants (Figure 7), while *arf2-7 abi3-1*, *arf2-7 abi4-1* and *arf2-7 abi5-1* had an ABA-sensitive phenotype similar to *arf2-7* mutant, suggesting that the *arf2* effect on root growth inhibition by ABA requires the canonical ABA signaling pathway that can be blocked by the dominant *abi1* or *abi2* mutation, but not by *abi3-1*, *abi4-1* or *abi5-1* mutation. Gene expression analyses in these *abi* mutants indicated that the induction of *ARF2* transcripts by ABA treatment was impaired by *abi1-1* mutation, partially reduced by *abi2-1* mutation, but not affected by *abi3-1*, *abi4-1* or *abi5-1* mutation (Figure 7B). Furthermore, *ARF2* induction by ABA was not changed in auxin receptor quadruple mutant *tir1 afb1 afb2 afb3* [36] (Figure 7C), indicating that *ARF2* expression is specially regulated by ABA signaling pathway.

### ABA treatment altered the auxin distribution or auxin signal in the primary root tips

**Figure 8:**
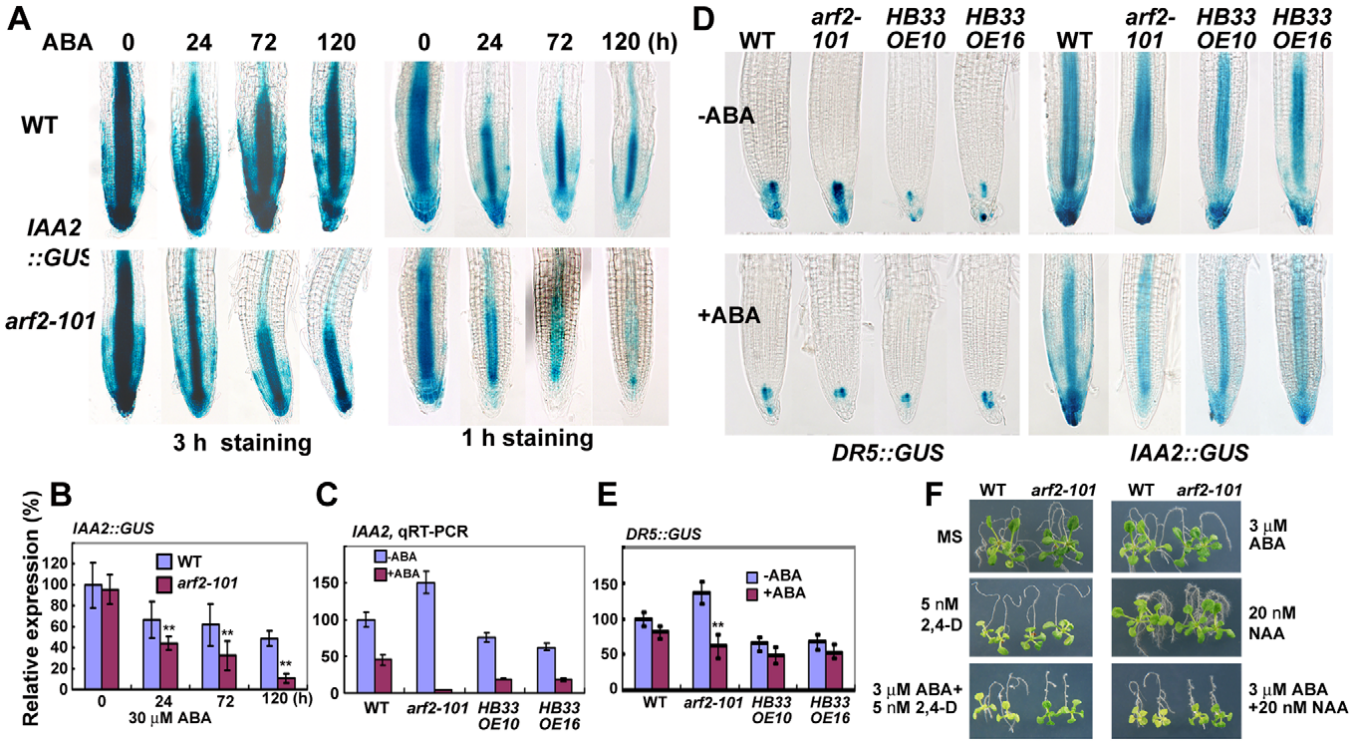
ABA treatment altered the auxin distribution or auxin signal in the root tips. (A) Promoter *IAA2::GUS* expression patterns in the wild type (WT) and *arf2-101*. Five-day-old seedlings were transferred to MS medium or MS medium containing 30 µM ABA and treated for different times before GUS staining. GUS staining was performed for 3 h and 1 h in order to see the relative GUS expression. (B) Relative expression of GUS by the intensity of GUS coloration. About 10 root tips were measured. The relative intensity without ABA treatment in the wild type is considered as 100%. (C) The relative expression of *IAA2* by qRT-PCR. Total RNAs extracted from root tips (about 1 cm) of WT, *arf2-101*, *HB33 OE10* and *OE16* seedlings treated with 30 µM ABA for 36 h were used for qRT-PCR. *ACTIN* was used as a control. Three biologically independent experiments were done with similar results. The results shown were from one experiment with triple technical repeats. Values are means ±SD. (D) *DR5::GUS* and pro*IAA2::GUS* expression patterns in WT, *arf2-101* as well as two *HB33* overexpressing lines *OE10* and *OE16* without or with ABA treatment. Five-day-old seedlings were transferred to MS medium containing 30 µM ABA for 36 h before GUS staining (*DR5::GUS* staining for 8 h, pro*IAA2::GUS* staining for 1 h). (E) Relative expression of GUS by the intensity of GUS coloration as determined in (B). 10 root tips were measured. The relative intensity without ABA treatment in WT is considered as 100%. (F) WT and *arf2-101* exhibited similar response to NAA and 2,4-D or a low concentration of ABA. Combining 2,4-D or NAA with ABA increased the root growth sensitivity more in *arf2-101* than in WT.

As ARF2 is an auxin response factor, and auxin and ABA have cross talk, we want to know whether the auxin components are changed under ABA treatment in *arf2* mutants. We first used the auxin responsive marker *IAA2::GUS* whose expression is closely related to endogenous auxin [37]. As shown in Figure 8A, under normal condition, there was no much difference of GUS staining (in cells of the QC, columella and the provascular tissue) between *arf2-101* and the wild type. ABA treatment reduced *IAA2::GUS* expression in both *arf2-101* and the wild type, but *IAA2::GUS* expression was reduced more in *arf2-101* than in the wild type at different times. *IAA2* transcripts by quantitive RT-PCR and the GUS intensity quantified using Adobe Photoshop CS (Adobe Systems Inc.; San Jose, CA, USA) software were consistent with *IAA2::GUS* expression pattern (Figure 8B, 8C). It appears that relative higher GUS staining was observed around QC and columella stem cells than other cells in *arf2-101* (staining for 1 and 3 hr), but not in the wild type. We further checked the expression of *DR5*, a synthetic promoter with AuxREs that reports auxin response [38]. We introduced *DR5::GUS* into *arf2-101* by crossing a *DR5::GUS* transgenic plant with *arf2-101*. Under normal condition, *arf2-101* exhibited more GUS staining than the wild type in root tips [in QC cells, columella stem cells, differentiated columella and weak expression in some vascular cells (Figure 8D, 8E, −ABA), suggesting that ARF2 negatively regulates *DR5::GUS* [23]. ABA treatment reduced GUS expression in both *arf2-101* and the wild type. However, we always observed that after ABA treatment, *DR5::GUS* expression was highly accumulated around QC center, columella stem cells, differentiated columella cells and weak expression in some vascular cells adjacent to the QC in the wild type (Figure 8D, +ABA), but GUS staining in the *arf2-101* was not so widely. In *arf2-101*, GUS staining was highly accumulated around QC and columella stem cells, but neither in vascular cells adjacent to the QC nor in differentiated columella cells (Figure 8D, +ABA). The results suggest that ABA treatment reduces the whole auxin accumulation or auxin response in the root tips of *arf2-101* and the wild type, but the relative auxin distribution or auxin signal in the QC and columella stem cells was higher or stronger than other cells in *arf2-101*, but not in the wild type.

We also introduced *DR5::GUS* or pro*IAA2::GUS* into two *HB33* overexpressing lines, *OE10* and *OE16*. In *HB33* over-expressing plants, *DR5::GUS* expression was lower than that in the wild type or *arf2-101* under normal condition (Figure 8D and 8E). ABA treatment did not apparently reduce the expression of *DR5::GUS* in *HB33* over-expressing plants (Figure 8D and 8E), but made *DR5::GUS* accumulated around QC and columella stem cells similar with the GUS distribution in *arf2-101*. ABA treatment reduced the expression of pro*IAA2::GUS* in *HB33* over-expressing plants to a level that was higher than that in *arf2-101*, lower than that in the wild type, which is consistent with *IAA2* transcripts quantified by qRT-PCR (Figure 8C). These results indicate that *HB33* over-expressing plants exhibit similar regulation on the expression pattern of *DR5::GUS* and *IAA2* as *arf2* mutants.

As ABA treatment reduces auxin accumulation or auxin signal, we want to know whether the *arf2-101* sensitivity is due to reduced auxin. We tested the sensitivity of *arf2-101* mutant to supplied auxin 1-napthalene acetic acid (NAA) and 2,4-dichlorophenoxy acetic acid (2,4-D). NAA can diffuse through the plasma membrane without the help of auxin carriers, while 2,4-D needs auxin carriers to penetrate the plasma membrane [39]. A low concentration of ABA combined with a low concentration of either 2,4-D or NAA inhibited the primary root growth of both the wild type and *arf2-101* more than the same concentration of only ABA, 2,4-D or NAA (Figure 8F). Root growth inhibition by ABA plus auxin was greater in *arf2-101* than in the wild type. The results indicate that ABA and auxin have a synergistic effect on inhibiting root growth, and the root ABA sensitivity in *arf2-101* is not due to the reduced auxin.

### Auxin facilitators are involved in ABA response

**Figure 9:**
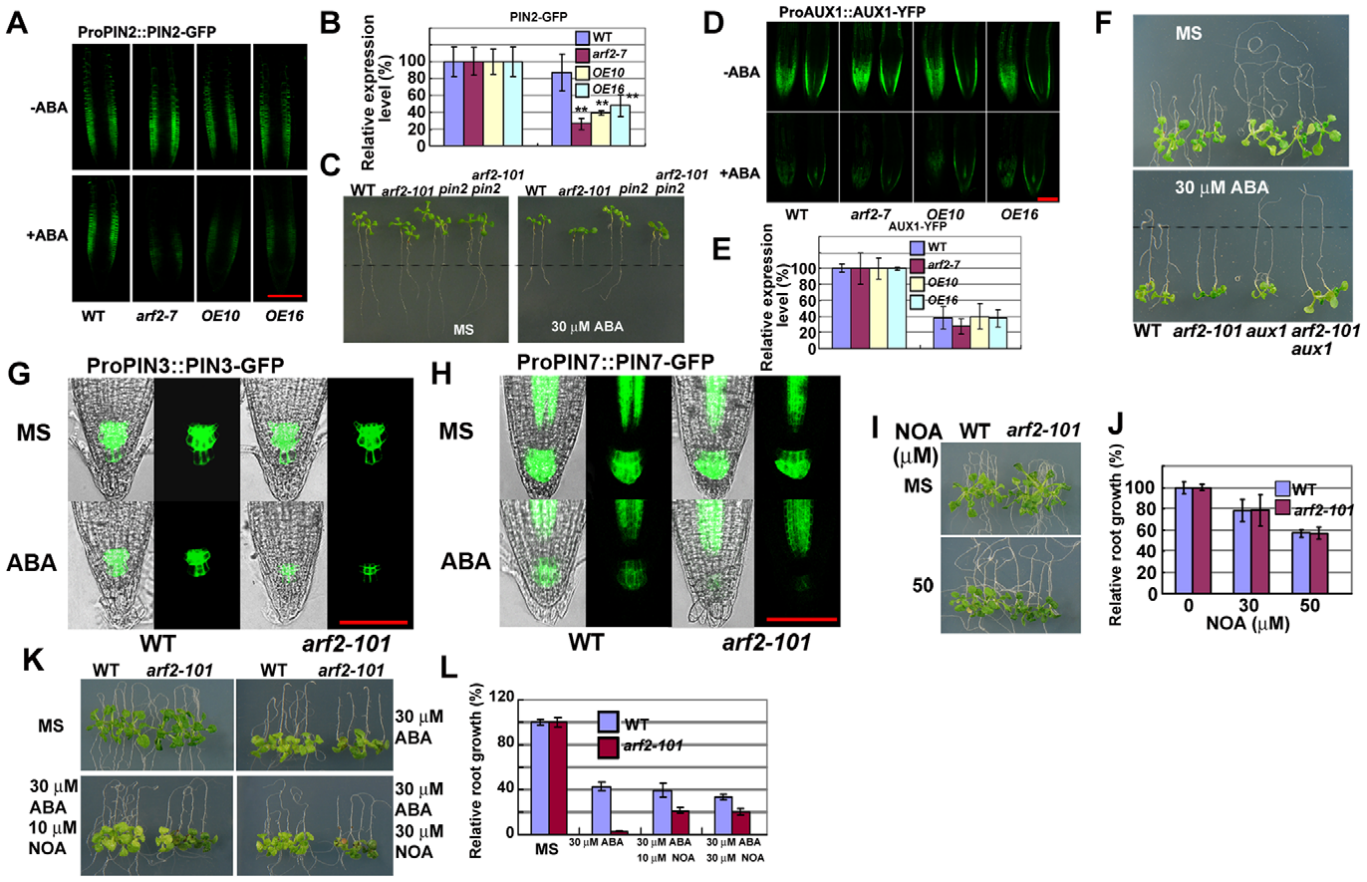
Auxin transport contributes to ABA inhibition of root growth. Transgenic plants carrying promoter *PIN2::PIN2-GFP* or promoter *AUX1::AUX1-YFP* were crossed with *arf2-101*, *HB33-OE10* or *HB33-OE16*, and homozygous plants of *arf2-101*, *HB33-OE10* or *HB33-OE16* with different fused GFP/YFP were identified by PCR and the offspring plants were analyzed. Five-day-old seedlings were transferred to MS medium containing 30 µM ABA for another 36 h before GFP/YFP images were captured with a confocal microscope at the same settings to enable comparison of image strength. 7–8 seedlings were examined for each sample, and similar results were obtained for each seedling within a sample. Values are means ±SD, **p<0.01. Bar = 100 µm. (A) promoter *PIN2::PIN2-GFP* (B) Relative expression of PIN2 by GFP intensity. (D) Promoter *AUX1::AUX1-YFP*. (E) Relative expression of AUX1 by YFP intensity. (C) Root growth phenotypes of the wild type (WT), *arf2-101*, *pin2*, and *arf2-101 pin2* seedlings. Five-day-old seedlings grown on MS medium were transferred to MS medium containing 0 and 30 µM ABA and were grown for 7 days before being photographed. (F) Root growth phenotypes of WT, *arf2-101*, *aux1*, and *arf2-101 aux1* seedlings. Five-day-old seedlings grown on MS medium were transferred to MS medium containing 0 and 30 µM ABA and were grown for 7 days before being photographed. (G) Pro*PIN3::PIN3-GFP* expression in the wild type and *arf2-101* with or without 30 µM ABA treatment for 36 h. Bar = 100 µm. (H) Pro*PIN7::PIN7-GFP* expression in the wild type and *arf2-101* with or without 30 µM ABA treatment for 36 h. Bar = 100 µm. (I,J) Effects of different concentrations of the auxin carrier influx inhibitor 1-naphthoxyacetic acid (NOA) on the root growth of WT and *arf2-101*. 30 seedlings were measured in each experiment. Three independent experiments were done. (K,L) NOA partially releases the ABA sensitivity of *arf2-101* under ABA treatment. Relative root growth of seedling treated with ABA, ABA plus NOA. About 30 seedlings were measured. Three independent experiments were done.

Because auxin facilitators affect auxin distribution and thereby mediate root meristem patterning [34], we examined whether PIN2, and AUX1 are involved in ABA mediating root growth. PIN2 is an auxin efflux carrier, and AUX1 is an auxin influx carrier [40]–[42]. First, we checked expression levels in the *arf2* mutant by using *PIN2* or *AUX1* promoter derived PIN2-GFP or AUX1-YFP transgenic plants. Interestingly, ABA treatment did not change the PIN2-GFP level so much in the wild type (Figure 9A, 9B), but greatly decreased the PIN2-GFP protein in the *arf2-101* mutant as well as *HB33-OE10* and *-OE16* (Figure 9A, 9B). PIN2 is a major regulator of the basipetal auxin transport that controls root meristem cell division [40]. We hypothesize that if the reduced PIN2 expression is the reason for ABA sensitivity, then *pin2* mutants should be sensitive to ABA treatment as *arf2-101*. However, *pin2* mutants exhibited similar ABA sensitive phenotypes as wild type in root growth, while *arf2-101 pin2* double mutants showed similar ABA sensitive phenotype as *arf2-101* in root growth (Figure 9C), indicating that the decreased PIN2 observed in *arf2-101* should be resulted from the ABA hyper-response, but is not the reason for ABA sensitivity.

PIN2 and AUX1 overlap for the basipetal auxin transport through the outer root cell layers. Besides, AUX1 is also responsible for the phloem-based auxin transport from source leaves to the root basal meristem [43]. For AUX1 expression, we also included *HB33* overexpressing lines. ABA treatment decreased the AUX1 to the similar level in the wild type, *arf2-101* and two *HB33* overexpressing lines (Figure 9D, 9E). In order to know whether auxin transported from phloem affects ABA sensitivity in root tips or not, we examined *aux1* mutant. *aux1* mutant showed more resistant to ABA in root growth than the wild type (Figure 9F). *arf2-101 aux1* double mutant showed phenotype similar to *aux1* that is more resistant to ABA than the wild type in root growth (Figure 9F).

Several auxin efflux carrier PINs (PIN3, PIN4 and PIN7) are expressed in QC and columella stem cells to regulate cell division and cell expansion in the primary root [34]. We used *PIN3* or *PIN7* promoter derived PIN3-GFP or PIN7-GFP to check their expression. Without ABA treatment, *arf2-101* and the wild type exhibited the similar expression level of *PIN3-GFP* or *PIN7-GFP*. ABA treatment reduced the expression of *PIN3-GFP* or *PIN7-GFP* in both the wild type and *arf2-101*, but the expression of *PIN3-GFP* or *PIN7-GFP* was reduced more in *arf2-101* than in the wild type (Figure 9G, 9H). These results indicate that the reduced expression of *PIN3* and *PIN7* might lead to high accumulation of auxin in QC and columella stem cells and inhibit cell division.

NOA is an inhibitor of auxin influx carrier that phenocopies *aux1* [44]. NOA concentrations from 30 to 50 µM did not differently affect root growth between *arf2-101* and the wild type (Figure 9I, 9J). When different concentrations of NOA were added to the medium, the root growth inhibition by ABA was differentially released, i.e., the inhibition of *arf2-101* became similar to that of the wild type (Figure 9K, 9L). These results indicate that inhibition of root growth by ABA in *arf2-101* is rescued or alleviated by blocking AUX1 mediating auxin influx, suggesting that ABA mediating root growth is involved in auxin transport.

## Discussion

It has long been known that root growth is inhibited by high levels of ABA, but the molecular mechanism is poorly understood [1], [17]. In our studies, we have used the Columbia accession to study the molecular mechanism of how ABA regulates root growth, and we have identified several new root-sensitive mutants to ABA [18]–[20], suggesting that our screening is very powerful in identifying new components in ABA response pathway. In the current study, we provide several lines of evidence to show that *ARF2* and its regulated gene *HB33* are important players in ABA response which has cross talk with auxin response pathway in regulating plant growth.

ARF2 was originally identified as an ARF1 binding protein (ARF1-BP) [26], [38], and could bind to AuxRE target site [45], [46]. Later, ARF2 was isolated as a suppressor that regulates hypocotyl bending of the *hookless1* mutant (an ethylene-response mutant), which suggested that ARF2 has an important role in linking ethylene and auxin signaling in the apical hook [23]. ARF2 is a pleiotropic regulator that represses the expression of targeted genes to regulate plant development [21], [22], [47]. Furthermore, brassinosteroid-regulated BIN2 kinase can phosphorylate ARF2, which releases the ARF2 repression activity and thereby increases expression of auxin-induced genes [28]. Here, we found that the expression of *ARF2* is induced by ABA, and that ARF2 is a negative regulator in the ABA response pathway controlling seed germination and primary root growth. In contrast, no single *ARF* gene among the 23 *ARF* members is a positive regulator in the control of embryonic axis growth by ABA, probably because of functional redundancy among the genes [3]. These results suggest that ARF2 is a central integrator which connects auxin, ethylene, brassinosteroid and ABA signal pathway in controlling the growth and development of different organs and tissues [3], [17], [32], [48], [49].

In the auxin signaling pathway, Aux/IAA proteins dimerize with and inhibit the activities of ARF proteins at low auxin concentrations [50], [51]. At high auxin concentrations, Aux/IAA is degraded by the F-box protein TRANSPORT INHIBITOR RESPONSE1 (TIR1)/AUXIN SIGNALING F-BOX (AFB)-mediated ubiquitination, which releases the inhibition on the ARFs [51]. The released ARFs then target downstream genes. In *Arabidopsis*, the ARF family has 23 members exhibiting either positive or negative regulatory roles [52]. Study of regulation of *ARF10* mRNA stability by miR160 suggested that ARF10 mediates the link between ABA and auxin responsiveness during and after seed germination [53]. Although ARFs are important in plant growth and development, only some of the direct targets of ARFs have been identified [54]. Previous studies have indicated that *ARF2* expression is also regulated by miRNAs and/or tasiRNAs [55], [56]. Our data indicate that ARF2 is a transcriptional repressor that directly targets the promoter of *HB33* and suppresses the expression of *HB33*. Consistent with the induced expression of *ARF2* by ABA is the suppressed expression of *HB33* by ABA. Although many AuxREs exist in the genomic sequence, our ChIP analyses indicate that ARF2 can only bind to some specific promoters including *HB33* with AuxREs, suggesting that the targeted sites are determined by both ARF2 and its partner(s). *HB33*, however, is only one of the genes targeted by ARF2. Our data indicate that ARF2 is a negative regulator in ABA mediating seed germination and root growth as *arf2* mutants are sensitive to, and ARF2 over-expressors are resistant to ABA in both seed germination and root growth. In contrast, HB33 is a positive regulator in ABA mediating seed germination and root growth as showed by both *HB33* over-expressing and *HB33* RNAi study. Our results suggest that ARF2 regulates seed germination and root growth partially through direct repression of *HB33* in Arabidopsis. However, reducing the expression of *HB33* by RNAi could not rescue the ABA sensitive phenotype of *arf2* mutant, suggesting that other components besides HB33 are needed for controlling the ABA response in *arf2*.

Previous study indicates that ABA has cross talk with ethylene in regulating plant growth [23], [57], [58]. Ethylene insensitive mutant *ein2* is recovered as a mutant with enhanced response to ABA in seed germination (named *era3*) [57], and also from screening the suppressors and enhancers of *abi1-1* in seed germination [58]. Interestingly, EIN2 is a negative regulator in seed germination, but a positive regulator in root growth as the root growth of *ein2* is more resistant to ABA than that of the wild type [57], [58]. As mentioned above, ARF2 was isolated as a suppressor of the *hookless1* mutant [23]. *ABI3* encoding a transcriptional factor is induced by auxin in lateral root primordia [59]. The ABA-insensitive *abi3* mutant reduces while the ABA-hypersensitive *era1* mutant increases the number of lateral roots when exogenous auxin is applied [59], [60]. *ABI4* encodes another transcriptional factor that is up-regulated by cytokinin and ABA, but repressed by auxin in roots [61]. These results indicate that ABA and ethylene signal have a cross-talk with auxin signal.

However, the molecular mechanism of ABA inhibition of primary root growth is different from that of ethylene. Addition of ethylene reduces cell elongation, but does not affect cell division, and does not change *CYCB1;1* expression [32]. But, addition of ABA reduces cell division and greatly decreases *CYCB1;1* expression. Ethylene inhibits root growth by increasing auxin biosynthesis and enhancing the expression of *PIN2* and *AUX1* [32], [49], [62]. Nevertheless, ABA inhibiting root growth seems mainly through interfering with the distribution of auxin in root tips. ABI4 negatively regulates PIN1 and interferes with auxin distribution to control lateral root growth [61]. Here, we used two marker genes, *DR5::GUS* and pro*IAA2::GUS*, to examine the gene expression by ABA treatment. Under normal condition, *DR5::GUS* was expressed at a higher level in *arf2-101* than in the wild type, indicating that *arf2* mutation results in a stronger auxin response. Although ABA treatment greatly reduced *DR5::GUS* expression in both wild type and *arf2-101* mutant, interestingly, ABA treatment made *DR5::GUS* accumulated to a relative, but not absolute, higher level around QC center and columella stem cells than other cells comparing with that in normal condition in both wild type and *arf2-101*. Different from wild type, GUS staining was not observed in differentiated columella cells and some vascular cells in *arf2-101*, but could be observed in the wild type. ABA treatment also decreased the expression of endogenous auxin responsive marker pro*IAA2::GUS* in both wild type and *arf2-101* mutant, but more in *arf2-101* than wild type. Again, the relative GUS staining became stronger around QC center and columella stem cells in *arf2-101* by ABA treatment comparing with no ABA treatment. Nevertheless, the relative stronger GUS staining was not so apparent in the wild type. These results suggest that ARF2 mutation interferes with auxin distribution and the relative high auxin accumulation or auxin signal around QC and columella stem cells inhibits the cell division in the root tips. *HB33* overexpressing plants show the similar ABA sensitive phenotypes as *arf2-101*. Interestingly, ABA treatment leads to the similar expression patterns of both *DR5::GUS* and pro*IAA2::GUS* in *HB33* overexpressing plants as in *arf2* mutant. However, the expression of both *DR5::GUS* and pro*IAA2::GUS* is lower in *HB33* overexpressing plants than in *arf2-101*, suggesting that *arf2* mutation might regulate the expression of other genes besides *HB33*. Previous study indicate that mutations in some DNA replication related proteins such as DNA polymerase ε (ABO4) and DNA replication A2A (ROR1) or FAS1 lead to ABA hypersensitivity in root growth, suggesting that ABA signal might target DNA replication related proteins for inhibiting DNA replication and cell division [18]. A recent study also shows that ABA treatment reduces the phosphorylation level of DNA replication factor C, suggesting the importance of ABA signal transduction in modifying DNA replication related proteins [63].

Auxin distribution is determined by auxin transporters [39], [64]. Although ABA treatment reduced the expression of the auxin basipetal efflux transporter PIN2 to a lower level in *arf2-101* than wild type, our genetic analysis of *arf2-101 pin2* double mutant excludes the possibility of PIN2 involving in ABA inhibition of root growth of *arf2* mutant, which is different from its roles in mediating ABA repression of embryonic axis elongation under ABA treatment [3]. The reduced PIN2 might be caused by the low auxin or low auxin signal after ABA treatment as PIN2 expression is regulated by auxin homeostasis [34]. AUX1 is a basipetal auxin influx transporter pairing with PIN2, but at the same time it functions in transporting auxin via phloem from source leaves to the root basal meristem [43]. AUX1 expression in roots was decreased by ABA treatment, but AUX1 levels did not differ between the wild type and *arf2-101* mutant. Our genetic analysis indicates that AUX1 mutation was able to suppress the ABA sensitivity of root growth of *arf2-101*, suggesting that auxin transporting from leaves to root tips is important in ABA inhibition of root growth. Interestingly, tow auxin efflux carriers PIN3 and PIN7 are reduced more in *arf2* than in the wild type by ABA treatment. PIN3 and PIN7 are key transporters that direct the flow of auxin in root tip [65]. The reduced expression of PIN3 or PIN7 might lead to relative high accumulation of auxin in QC and columella stem cells, which might result in the inhibition of cell division. Although ABA treatment appears to reduce auxin biosynthesis or reduce the whole auxin signal (judged by reduced *IAA2::GUS* and *DR5::GUS*), our auxin feeding experiment indicates that auxin and ABA have a synergistic effect on inhibiting root growth, suggesting that the possible reducing whole auxin amount is not a factor for ABA sensitivity. These results further point out the importance of auxin distribution in ABA inhibiting root growth. Previous studies also indicate that ABA inhibits seedling growth through enhancing auxin signaling [3]. Mutations in some auxin components such as AXR2/IAA7 and AUX1 lead plants to be resistant to both ABA and auxin [3], [42], [66], [67]. This synergistic effect requires the canonical ABA signaling pathway, which is blocked by the dominant *abi1* or *abi2* mutation, but not by *abi3*, *abi4* or *abi5* mutation, indicating that the importance of early ABA signaling components in ABA inhibiting root growth.

## Materials and Methods

*Arabidopsis thaliana* (Columbia accession) was used unless noted. The plant materials used in this study were: *abi1-1* (Landsberg accession) [4], *abi2-1* (Landsberg accession) [68], *abi3-1* (Landsberg accession) [69], *abi4-1* (Columbia accession) [19], *abi5-1* (Columbia accession) [19], *tir1 afb1 afb2 afb3* quadruple mutant [36], pro*CYCB1;1::GUS* [18], *pin2_151A5.1/CS89461* [70], *aux1-2* [71], pro*DR5::GUS* [38], pro*IAA2::GUS* [62], pro*AUX1-AUX1:YFP* [43], and *proPIN2-PIN2::GFP* [72], pro*PIN3-PIN3:GFP* and pro*PIN7-PIN7:GFP* [34]. The T-DNA insertion mutant *arf2-7* (CS24601, *AT5G62000*), *arf1* (CS24599, *AT1G59750*), *arf6* (CS24606, *AT1G30330*), *arf21* (CS24621, *AT1G34410*) were obtained from the Arabidopsis Stock Center. *arf2-101* and *arf2-102* mutants were isolated from a screen as described previously [18]. *arf2-101* was crossed with Landsberg accession, and mutants from the F2 population were used for mapping (Table S2).

Seeds were sown onto plates containing MS medium supplemented with 3% (w/v) sucrose and 0.8% agar. After 2 d at 4°C, the plates were transferred to a growth chamber at 20°C with a 16-h light/8-h dark cycle. After 7 days, the seedlings were transplanted into soil and were grown in a greenhouse at 20°C under long-day (16-h light/8-h dark) condition.

### Transgenic plants

For construction of super promoter*-ARF2-flag* (in a modified pCAMBIA 1300 vector, superP3101), *ARF2* cDNA fragment was obtained using the following primers: 5′- CGCGGGCCCGTATGGCGAGTTCGGAGGTTTCAATG (containing the *Apa*I site) and 5′- CGGACTAGTAGAGTTCCCAGCGCTGGAC-3′ (containing the *Spe*I site). *ARF2* cDNA amplified from total RNAs isolated from seedlings was fused with a flag tag and constructed into a binary vector superP1300. For super P1300-*ATHB33: MYC*, *ATHB33* cDNA fragment was amplified by the specific primer: 5′-CGCGGGCCCCCATGGATATGAGAAGCCATGAAATGATAGAGAG-3′ (containing the *Apa*I site) and 5′-GGACTAGTGAGAGTAGTTGTTGGTGTTGGTGG-3′ (containing the *Spe*I site). The amplified cDNAs were verified by sequencing and were cloned into the binary vector super P1300. The *Agrobacterium* strain GV3101 carrying the constructs was transformed to *Arabidopsis thaliana* (Columbia) by the floral dip method. More than 10 independent transgenic lines were selected based on hygromycin B resistance in the T2 and T3 generations. The expression level of the gene in each line was analyzed by real-time PCR using specific primer pairs. Two independent lines were generally used for further study.

We constructed pGreen104-HY104 : HB33 vector to silence *HB33* gene expression by double-stranded RNAi. A *HB33* cDNA fragment which has low homologous sequence with other *HB* genes was chose and amplified using two primer pairs: RNAi-75240-F:5′- GCTGCA (*Pst*1) GAATTC (*Eco*R1) GGACGGCGTTGGAAGCTCG-3′, RNAi-75240-R:5′- CGGATC (*Bam*H1) CTCGAG (*Xho*1) CACCAGCTCCTTCTCCGCTTG -3′. In bacteria, the plasmid was selected by Kanamycin. The transgenic plants were selected by Basta. Homozygous lines were identified in T3 generation and used for further study.

### Phenotypic analyses

The root-bending assay was previously described [18]. Briefly, Seeds sown on MS plates were first kept at 4°C for 2 days and then transferred to a growth chamber at 22°C for 5 days. Seedlings were transferred to various media containing different plant hormones or chemicals; the seedlings were photographed after 7 days unless a different time period is indicated. Relative root growth represents the root growth of seedlings after treatment with ABA or other chemicals comparing with that without ABA or other chemical treatment.

### Purification of recombinant protein and electrophoretic mobility shift assay (EMSA)

The N-terminal of ARF2 which contains DNA binding domain was fused in frame with GST and expressed in *E. coli* BL21 cell line. The fused protein was induced by 0.2 mM isopropyl β-D-1-thiogalactopyranoside (IPTG), and incubated under 28°C for 6 h. The recombinant protein was purified by GST-agarose affinity. The electrophoresis mobility shift assay (EMSA) was carried out using the LightShift Chemiluminescent EMSA Kit (Pierce, 20148) according to the manufacturer's instructions. The biotin-labeled DNA fragments (5′-CCAAATTGTCTCTTATAATCCGATCTCAAAGGTTGGCTTGTTGGGAGCACTGAGACATCAAAC-3′, 5′-GTTTGATGTCTCAGTGCTCCCAACAAGCCAACCTTTGAGATCGGATTATA AGAGACAATTTGG-3′) and mutated DNA fragments (5′-CCAAATTGaCaCTTATAATCCGATCTCAAAGGTTGGCTTGTTGGGAGCACTGtGtCATCAAAC-3′, 5′-GTTTGATGACACAGTGCTCCCAACAAGCCAACCTTTGAGATCGGATTATAAGTGTCAATTTGG-3′) were synthesized, annealed and used as probes, and the biotin-unlabeled same DNA fragments as competitors in this assay. The probes were incubated with the N-ARF2-GST fused protein at room temperature for 20 mins in a binding buffer (5× concentration: 50 mM HEPES-KOH [pH 7.5], 375 mM KCl, 6.25 mM MgCl, 1 mM DTT, 0.5 µg/mL BSA, Glycerol 25%). Each 20 µl binding reaction containing 25 fmol Biotin-probe, 6 µg protein, and 1 µg Poly (dI·dC) was supplemented to the reaction to minimize nonspecific interactions. The reaction products were analyzed by 6.5% native polyacrylamide gel electrophoresis. Electrophoresis was performed at 120 V for about 1 h in TGE buffer (containing 12.5 mM Tris, 95 mM glycin, 0.5 mM EDTA, pH 8.3, precooled at −10°C). The DNA fragments on gel were transferred onto nitrocellulose membrane with 0.5XTBE at 100 V (∼380 mA) for 40 mins at 4°C. After cross-linking the transferred DNA to membrane, the membrane was incubated in the blocking buffer for 15 mins with gently shaking, then transferred to conjugate/blocking buffer by mixing 33.3 µl stabilized Streptavidin-Horseradish Peroxidase Conjugate with 10 ml blocking buffer according to manufacture's protocol (no detail information for the blocking buffer is provided in the kit). The membrane was washed 6 times, each for 5 mins with a washing buffer. Biotin-labeled DNA was detected by the chemiluminescent method according to the manufacture's protocol.

### Chromatin immuno-precipitation (ChIP) analysis

The transgenic lines over-expressing ARF2-Flag were used in this assay. ChIP was performed on 2-week-old seedlings growing on MS plates as described previously [73]. Flag tag-specific monoclonal antibody was used for ChIP analysis. Wild-type plants were treated in the same way and served as the control. The ChIP DNA products were analyzed by PCR using three pairs of primers that were synthesized to amplify about 200-bp DNA fragments in the promoter region or coding region of *HB33* or other genes used in ChIP analysis. The primer sequences were listed in Figure S3 (Table S1). The experiment was repeated three times, and similar results were obtained each time.

### RNA extraction and quantitative RT–PCR

Two-week-old seedlings on MS plates were used for extraction of total RNAs. Real-time RT-PCR was performed as described previously to determine the relative expression levels of *ARF2* or *HB33* [18]. The gene-specific primers used for real-time PCR were: RT-ARF2-F: 5′- CCTCATCCGAAGGATGCTCAA ACG -3′, RT-ARF2-R: 5′- GGAGCCATCAACTCTCCATTG AACTC -3′; RT-HB33-F: 5′-GGACAATCAAGCGGAGAAGGAGC-3′; RT-HB33-R: 5′-CTCCGATCTCGCCGCAGAATCTC-3′. All experiments were independently repeated at least three times, each with triplicates.

### Histochemical GUS analysis

The transgenic plants carrying pro*DR5::GUS*, pro*IAA2::GUS*, pro*CYCB1;1::GUS HB33-OE10* or *HB33-OE16* were crossed with the *arf2-101* mutant, and F2 seedlings were selected on kanamycin and transferred to soil after 1 week. Plants that showed the *arf2-101* growth phenotype were harvested and rechecked for other markers. Expression analysis of the GUS reporter gene was performed as described previously [18].

### Confocal microscopy

Five-day-old seedlings carrying pro*AUX1-AUX1::YFP*, pro*PIN2-PIN2::GFP*, p*roPIN3-PIN3:GFP* or pro*PIN7-PIN7:GFP* were transferred to MS medium supplemented with 30 µM ABA for another 36 h. The roots were examined by confocal microscopy and photographed with the same settings to enable comparison of image strength.

## Supporting Information

Figure S1 *arf2-101* shows the similar phenotypes as the wild type on MS medium containing different chemicals. 5-day seedlings grown on MS medium were transferred to the MS medium containing different concentrations of NaCl, mannitol, LiCl, 2,4-D, IAA, NAA, ACC, brassinosteroid (BR), coronatine, methyl-jasmonate (MeJA), gibberellin (GA3), or salicylic acid (SA), and cultured for 7 days before taking pictures.
(TIF)

Table S1 The primers used for ChIP.
(DOC)

Table S2 Primers used for map-based cloning.
(DOC)
