# Supplementary figures and images for: Auxin Response Factor2 (ARF2) and Its Regulated Homeodomain Gene HB33 Mediate Abscisic Acid Response in Arabidopsis
Source: PLoS Genet. 2011 Jul 14;7(7):e1002172. doi: 10.1371/journal.pgen.1002172 (PMC3136439; doi:10.1371/journal.pgen.1002172)

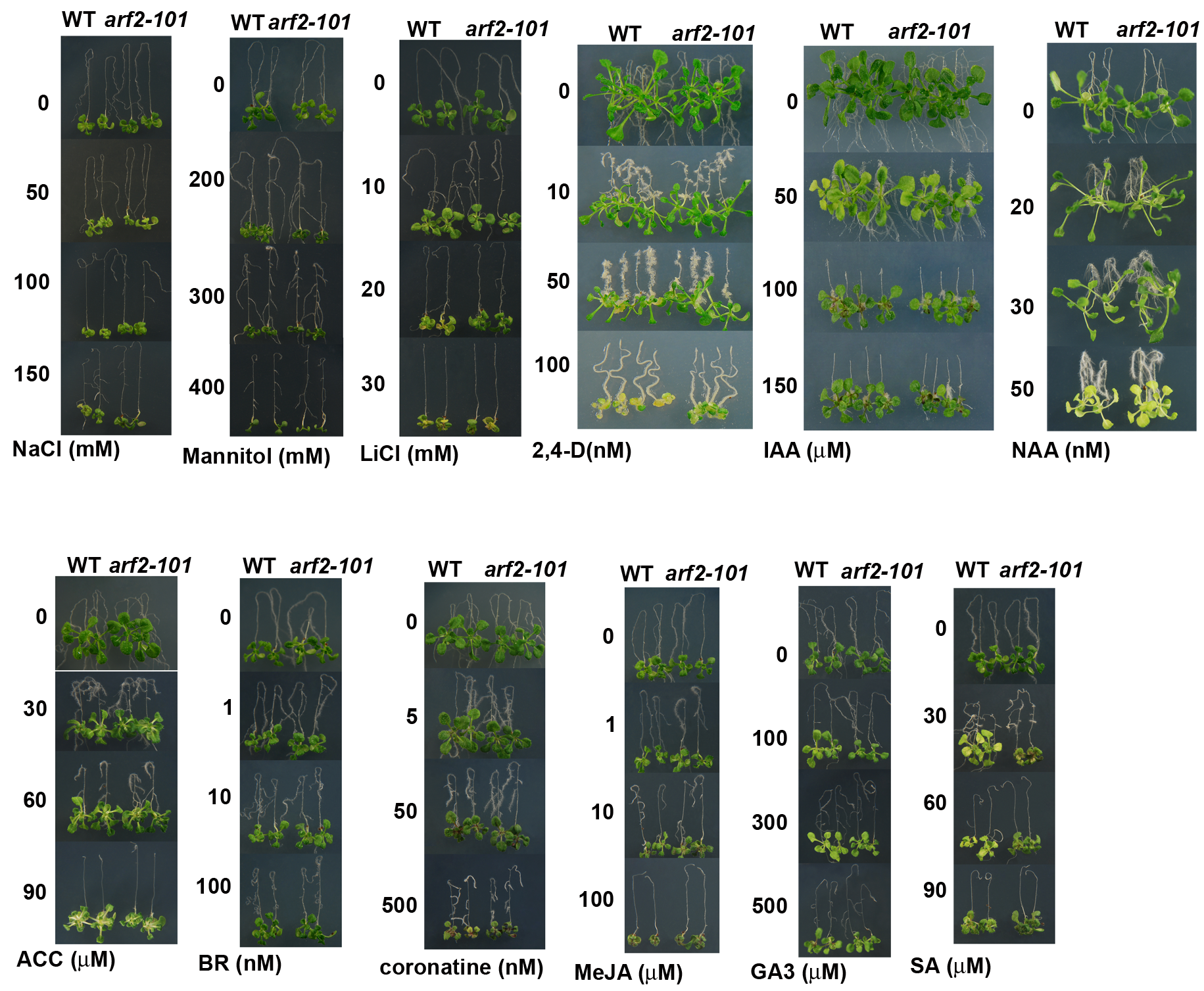

Supplement: Figure S1 — arf2-101 shows the similar phenotypes as the wild type on MS medium containing different chemicals. 5-day seedlings grown on MS medium were transferred to the MS medium containing different concentrations of NaCl, mannitol, LiCl, 2,4-D, IAA, NAA, ACC, brassinosteroid (BR), coronatine, methyl-jasmonate (MeJA), gibberellin (GA3), or salicylic acid (SA), and cultured for 7 days before taking pictures. (TIF) [file pgen.1002172.s001.tif]
