# Supplementary material for: Auxin Response Factor2 (ARF2) and Its Regulated Homeodomain Gene HB33 Mediate Abscisic Acid Response in Arabidopsis
Source: PLoS Genet. 2011 Jul 14;7(7):e1002172. doi: 10.1371/journal.pgen.1002172 (PMC3136439; doi:10.1371/journal.pgen.1002172)
Supplement: Table S1 — The primers used for ChIP. (DOC) [file pgen.1002172.s002.doc]

| **Table S1, Primers used for ChIP** | |
| --- | --- |
| Primer name | Primer sequence |
| CHIP-HB33- F1 | 5'- TGCATCAGTTTCATGCGAGTTACAC -3' |
| CHIP-HB33- R1 | 5'- GAGTCCGATAGAGTGTCCTCCTG -3' |
| CHIP-HB33- F2 | 5'- CATCTCTCACAAAGGGCTCAGC -3' |
| CHIP-HB33- R2 | 5'- GCTCAAAGCTTTTTGCTTGTGAAGG -3' |
| CHIP-HB33- F3 | 5'- CACCACCACAAG TATGGAATG AGTCC -3' |
| CHIP-HB33- F3 | 5'- GTCCATCATCCTCTCCTTCTGATCC -3' |
| Actin-F | 5'- TATGGTCAAGGCTGGGTTCG-3' |
| Actin-R | 5'- CCATGCTCGATGGGGTACTT-3' |
| CHIP-At4g33680-F | 5'- CCTTGGAGTAGATGGATCTGGAAACTG -3' |
| CHIP-At4g33680-R | 5'- CCTTGCTGAACACCAAGAACTGTAG -3‘ |
| CHIP-SAUR-15-F | 5'- GGTACACAACTTCATGTCCCTACATAATCT -3' |
| CHIP-SAUR-15-R | 5'- TGCTTAGCACCCAAGAAACTCCTC -3' |
| CHIP-GH3.1-F | 5'- GGA AGG AGA CTG GAA AGA CAC TTG CTG G -3' |
| CHIP-GH3.1-R | 5'- CAT GCG ACC TCC TCC TAT CGT GC -3' |
