# Supplementary material for: Auxin Response Factor2 (ARF2) and Its Regulated Homeodomain Gene HB33 Mediate Abscisic Acid Response in Arabidopsis
Source: PLoS Genet. 2011 Jul 14;7(7):e1002172. doi: 10.1371/journal.pgen.1002172 (PMC3136439; doi:10.1371/journal.pgen.1002172)
Supplement: Table S2 — Primers used for map-based cloning. (DOC) [file pgen.1002172.s003.doc]

Table S2 The primers used for map-based cloning

| Primer name | Primer sequence |
| --- | --- |
| F15L12-F | 5'- TGCCGGCTTCGAGTTTCCAATCATC -3' |
| F15L12-R | 5'- TGCCGTCGGTATCGTCAATCATCACC -3' |
| MM19-F | 5'- GGAGTATTTTGTGTGGTGAGAAGAGTG -3' |
| MM19-R | 5'- CAGATGCCATCTCTCATGCATGC -3' |
| MQB2-F | 5'- GTAAAATGAAAACTAAAAGGCGACTACTAGC -3' |
| MQB2-R | 5'- GCAAAATATTATGATCTTGCCATTTATTTGGTC -3' |
| MBK5-F | 5'- CAG AAGCTTTTTGGCCCATCTAGAG -3' |
| MBK5-R | 5'- CAAGATTTGCATGCATAGATCCAATCC -3' |
